# Editorial: Recent advances in discovering molecular targets for cancer therapy

**DOI:** 10.3389/fmed.2024.1403466

**Published:** 2024-05-08

**Authors:** Zhaoqi Yang, Teng Wang

**Affiliations:** ^1^School of Life Sciences and Health Engineering, Jiangnan University, Wuxi, China; ^2^Department of Oncology, Affiliated Hospital of Jiangnan University, Wuxi, China

**Keywords:** molecular targets, cancer therapy, immunotherapy, biomarkers, precise

As one of the main factors resulting in high morbidity and mortality worldwide, cancer is threatening human health constantly. 19.3 million cancer-related cases were recorded globally with a 51.8% death rate according to a study of the Global Cancer Observatory in 2020 (1, 2). Clinically, conventional therapeutic methods, for example, surgery, radiation therapy, and chemotherapy, have been applied in sole or combination (3). However, the achievement of a desirable curative effect could be potentially hindered by the non-selective action of routine therapies toward normal cells. Consequently, there remains an urgent need to develop targeted cancer treatment with the characteristic of enhanced cell specificity (4). Over the past decade, rapid progress in novel therapeutic strategies coupled with tumor-targeted candidates, containing neoantigens, hormonal agents, molecularly targeted agents, immune checkpoint inhibitors, and selective cytotoxic treatment, has offered new horizons for cancer-related molecular target discovery (5). Moreover, accumulating evidence indicates that targeting hypoxia-inducible factor-1 (HIF-1), Janus Kinase (JAK) family, tyrosine kinase, or epidermal growth factor receptor (EGFR) pathway may supplement a versatile and promising toolbox for improved survival benefits during the therapeutic process (4, 6). Despite the fruitful anticancer effectiveness of the above targets, these approaches are restricted in patients harboring some mutations or drug resistance. Hence, an in-depth understanding of recent advances in molecular target discovery may inspire the further development of precise and personal medicine in future cancer treatment.

Fitzsimmons et al.'s article primarily summarized the relevant trial data of immune checkpoint inhibitors (ICPI) and investigated the potential use of programmed death-ligand 1 (PD-L1) expression as a biomarker. In detail, a systematic review and meta-analysis, including 46,510 participants, were employed to explore the relationship between PD-L1 and ICPI biomarkers. Accordingly, a preliminary conclusion can be drawn that ICPI could serve as a tumor-agnostic therapy successfully, given its improved survival outcomes in all cancer types. Notably, a potentially problematic issue may be found in PD-L1 immunohistochemistry when it comes to directing ICPI use. Moreover, further research should be accomplished on other biomarkers such as tumor mutational burden and mismatch repair status.

Considering the expression of biomarkers is potentially related to the pan-cancer analysis, this research holds considerable scientific value and clinical significance for elucidating the link between PD-L1 expression and ICPI treatment efficacy, thereby offering a theoretical foundation for cancer diagnosis based on molecular characteristics. Collectively, to better assess the efficacy of ICPI-based solid cancer, the most comprehensive discussion concerning PD-L1 was presented, promoting future innovation and improvement in discovering targeted cancer therapies.

Guo et al. reported that tumor initiation and progression are influenced by a diverse array of cellular and non-cellular components that comprise the tumor microenvironment (TME). Recently, among various cancer targets, TME has also been deemed a promising therapeutic target for tumor treatment attributed to its key determinant role in cancer prognosis and outcome. According to the study, diverse strategies associated with TME have been discovered, consisting of targeting the extracellular matrix such as the CD44 receptor, targeting tumor hypoxia alone with key endogenous hypoxia markers like HIF-1 and carbonic anhydrase IX (CAIX), targeting tumor-promoting chronic inflammation with the COX-2 pathway, and targeting the tumor immune system. Furthermore, to validate the efficacy of the above factors and pathways that target TME, numerous clinical trials have been conducted extensively in recent decades, for example, bortezomib and bevacizumab. The scientific value of this review article lies in the molecular basis of the interaction between tumor cells and the TME and in emphasizing the potential value of TME-related anti-cancer agents, improving and refining the drugs in current clinical trials with elevated clinical confidence. Taken together, the established targeting methods and achieved encouraging results have offered a novel perspective for developing TME-induced anti-cancer therapeutic strategies. Liu et al. proposed an innovative lung adenocarcinoma (LUAD) identification methodology by CT-based radiometric phenotypes with the merits of non-invasive characteristics. According to the study, only a tiny percentage of patients with unique genetic traits or tumor-immune microenvironments can successfully benefit from immunotherapy or targeted therapy with the prerequisite of obtaining tissue specimens. To further select patients who benefited from targeted therapy, a convenient and efficient tissue sample identification method is urgently needed. Specifically, through a retrospective study of 288 LUAD patients in pathological diagnosis with profiled radiometric features and genomic data, it was found that four radiometric phenotypes can be classified on the basis of consensus clustering, demonstrating the potential application in providing individualized treatment for patients diagnosed with LUAD. This original research is closely related to our topic since it aims to providing the concrete evidences between effective LUAD patients identification and CT-based radiomic phenotypes in non-invasively way. By understanding the CT-based radiomic phenotypes in depth, more advanced and precise diagnostic options without invasive treatment can be offered for patients. In future clinical environments, it may complement conventional imaging by integrating radiogenomic into current processes, enabling patients with LUAD to get individualized treatment.

Dou et al. address the complications of chimeric antigen receptor T (CAR-T) cell immunotherapy in the context of promising treatment applications for hematological malignancies highlighting the employment of interleukin 6 (IL-6) monoclonal antibody to reduce the incidence of cytokine release syndrome (CRS). It is worth noting that the potential impact of administering IL-6 monoclonal antibody prophylactically prior to CAR-T treatment on the occurrence of CRS remains unclear. Therefore, given CRS is the major side effect of CRT-T therapy, meta-analysis in this research was employed to illustrate the relationship between the incidence of CRS and preventative use of IL-6 monoclonal antibody. Through the meta-analysis of a total of 111 articles, it was found that the incidence of CRS can be decreased by IL-6 monoclonal antibody at an 8 mg/kg dose 1 h prior to CAR-T cell infusion. Despite extensive experimental study is still required to better clarify the correlation between IL-6 cytokine levels and CRS occurrence, it provides clinical work with a direction of reduced side effects. Importantly, this research encompasses two trials with 37 participants that highlight the function of prophylactic use of interleukin 6 monoclonal antibody, it may offer a future direction for clinical process with minimized CRS side effects during CAR-T cell immunotherapy. Qiu et al. investigated the specific relationship between epidermal growth factor receptor (EGFR) mutation status and non-small cell lung cancer (NSCLC) patients such as its imaging, demographic, and ultimately pathologic characteristics. Briefly, a thorough literature search consisting of a total of 1,740 articles from various databases was carried out, aiming to perform a systematic review and meta-analysis of the above relationship. Interestingly, as for female higher-age patients with enlarged tumor diameter, an increased occurrence of EGFR mutations could be observed according to the meta-analysis, benefiting the assessment of molecular pathological changes in stage IA NSCLC. Above all, a convenient protocol was presented by using the presence status of ground glass opacity and final histologic classification, initiating better-personalized treatment in a cost-effective manner. In particular, a deep understanding of EGFR mutation factors will greatly help thoracic surgeons make initial assessments of early-stage lung nodules and reduce the financial burden to a large extent, ensuring the patients can receive an effective diagnosis promptly.

In conclusion, different novel targets that interacted with clinical diagnostic therapeutic and preventive approaches have recently been further developed due to advances in molecular biology, genome-wide analysis, and a deeper understanding of cancer intricacy. Accordingly, these advanced findings have bolstered the understanding of cancer-related molecular targets and offer novel perspectives and tools for early detection and treatment. In order to encourage innovation and advancement in individualized tumor treatment, future research should carry out an investigation into these possible biomarkers and therapeutic targets.

## Author contributions

ZY: Writing – original draft, Writing – review & editing. TW: Writing – review & editing.
